# Mouse Adipose Tissue Protein Extraction

**DOI:** 10.21769/BioProtoc.3631

**Published:** 2020-06-05

**Authors:** Yu A. An, Philipp E. Scherer

**Affiliations:** Touchstone Diabetes Center, Department of Internal Medicine; Department of Cell Biology, The University of Texas Southwestern Medical Center, Dallas, USA

**Keywords:** Obesity, Adipose Tissue, Protein, Lipid Contamination, Mouse, Metabolism, Time and Cost Effective

## Abstract

As obesity becomes a global epidemic, the metabolism research field is increasingly focusing on studying the physiological and pathological roles of adipose tissues (AT). However, extracting proteins from AT is challenging due to abundant fat content of intracellular lipid droplets. Several commercial kits for extraction of AT proteins are available, as are protocols (such as the RELi protocol as well as other protein precipitation protocols). The protocols have been introduced to improve the quality and yield of extractions, but these methods either increase the cost or involve multiple steps. Herein, we describe a detailed protocol for mouse AT protein extractions based on our daily laboratory practice. This protocol requires only very common reagents and instruments, and can be completed in 90-120 min and provides good recovery of total protein content. Thus, this protocol is an economically attractive, time-saving and efficient way to extract proteins from the AT.

## Background

Investigating adipose tissue (AT) to address issues related to the pathophysiology of obesity frequently involves analyzing AT proteins. However, lipid contamination in AT protein samples profoundly affects the accuracy of protein quantification, the quality of Western Blotting images, and the sample processing for downstream applications. To minimize lipid contamination, commercial kits have been developed, such as the Minute^TM^ Total Protein Extraction Kit for Adipose Tissues/Cultured Adipocytes (Invent Biotechnologies Inc., 2017). More comprehensive protocols aim to reduce lipid content, such as the Removal of Excess Lipids (RELi) protocol (Diaz Marin *et al.*, 2019) and the trichloroacetic acid (TCA)-based protein precipitation method (Benabdelkamel *et al.*, 2018). Although improved extraction quality has been demonstrated utilizing the above procedures, the disadvantages of these methods are also obvious: taking advantage of commercially available kits involves considerably higher costs; comprehensive protocols involve multiple steps, consume much more time, and might compromise total yields.

Herein, we aim to describe a detailed mouse AT protein extraction protocol that has been widely applied in our laboratory (An *et al.*, 2017 and 2019), not only to overcome the lipid contamination problem, but also to represent an easier to handle as well as a time and cost effective way to achieve high yields of total protein extraction from mouse AT. Our protocol requires very common reagents and instruments, and is therefore budget-friendly and can be routinely performed in most laboratories, even in those new to adipose biology research. The whole process of extracting proteins from frozen adipose tissues takes approximately 90-120 min, and is therefore a time effective approach. Furthermore, this protocol ensures maximal depletion of lipid contamination by removing the lipid layer before adding detergents and repeated centrifugation after tissue lysis. Last but not least, the protocol allows to recover a total of ~2.0 mg protein from ~100 mg white adipose tissues (~4.0 mg protein from ~100 mg brown adipose tissues), which is sufficient for several rounds of Western Blot analyses as well as other applications. Thus, the protocol below is a cost effective, time-saving and efficient protocol to extract proteins from different fat pads.

## Materials and Reagents

1. Safe-Lock tubes (2.0 ml, Eppendorf, catalog number: 022363352; 1.5 ml, Eppendorf, catalog number: 022363204)
2. Pipette tips (Rainin, catalog number: 17005873, 20 μl; catalog number: 17005875, 250 μl; catalog number: 17007090, 1,000 μl)
3. Stainless Steel Beads, 5 mm (Qiagen, catalog number: 69989), store at room temperature
4. Tris Hydrochloride (HCl), 1 mol/L solutions (pH = 8.0) (Fisher Scientific, catalog number: BP1758-500), store at room temperature
5. Ethylenediaminetetraacetic acid (EDTA) (Sigma-Aldrich, catalog number: E9884), store at room temperature
6. Sodium Chloride (NaCl) (Fisher Scientific, catalog number: S271-10), store at room temperature
7. Triton X-100 (Integra Chemical Company, catalog number: T756.30.30), store at room temperature
8. cOmplete Mini, EDTA-free protease inhibitor cocktail tablets (Roche, catalog number: 11697498001), store at 2-8 °C
9. Optional: phosphatase inhibitors if phosphorylated proteins will be detected (Phosphatase Inhibitor Cocktail 2: Sigma-Aldrich, catalog number: P5726; Phosphatase Inhibitor Cocktail 3: Sigma-Aldrich, catalog number: P0044), store at -20 °C
10. Pierce ^TM ^BCA Protein Assay Kit (Thermo Scientific, catalog number: 23225), store at room temperature
11. Liquid nitrogen
12. RIPA buffer (see Recipes)

## Equipment

1. Qiagen TissueLyser II homogenizer (Qiagen, catalog number: 85300)
2. Thermo Scientific microcentrifuge (refrigerated) (Thermo Scientific, model: Sorvall^TM^ Legend^TM^ Micro 17R, catalog number: 75002404)

## Procedure

- Mouse adipose tissue harvesting
Harvest adipose tissues [including inguinal/subcutaneous white fat pads (sWAT), visceral/epididymal white fat pads (eWAT), interscapular brown fat pads (BAT), *etc.*, as shown in Figure 1, details see article (Mann *et al.*, 2014)] and snap freeze in liquid nitrogen.
  **Figure 1.**
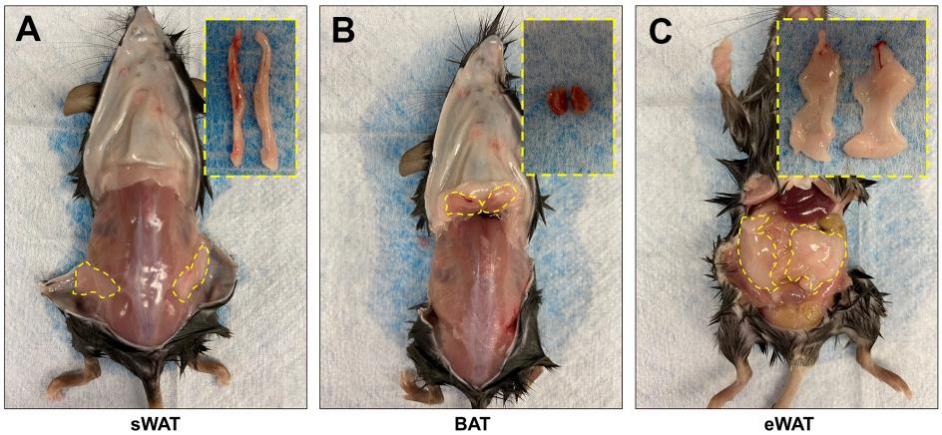
Mouse fat pads harvested in different locations. A. Subcutaneous WAT (sWAT). B. Interscapular brown AT (BAT). C. Epidydimal WAT (eWAT).
- Tissue homogenizing
  1. Add 0.5 ml RIPA buffer without Triton X-100 (~100 mg adipose tissue in a 2.0-ml tube with one stainless steel bead) with protease inhibitor and homogenize using the TissueLyser II (Qiagen) at the highest frequency for 3-5 min until clear. Keep on ice.
  2. Spin down at 6,000 *× g* for 15 min at 4 °C.
  3. Carefully remove the fat cake (refers to the white lipid layer on top of the aqueous layer as shown in Figure 2) and resuspend the loose pellet.
*Note: Alternatively, use the pipette tip to penetrate the fat cake and transfer the solution and the pellet to a new 1.5-ml tube.*
    **Figure 2.**
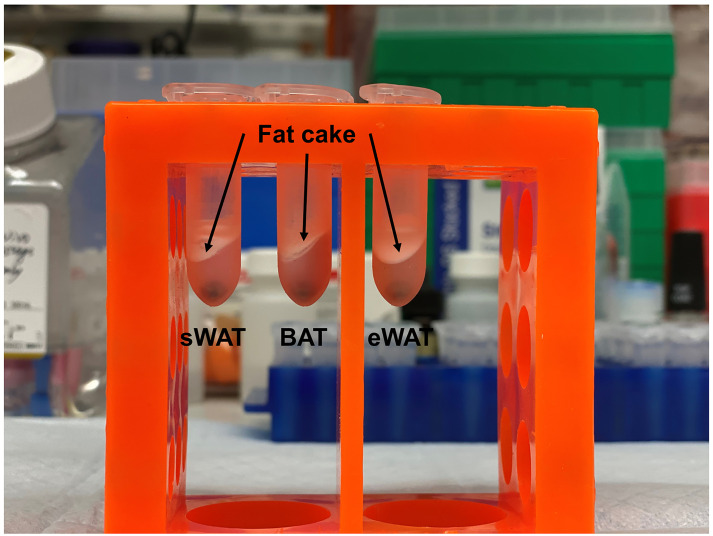
Fat cakes in different samples. After homogenization and centrifugation, fat cakes, indicated by arrows, are the white lipid layers on top of the aqueous layers in sWAT, BAT and eWAT samples.
- Tissue lysis and centrifuging
  1. Add Triton X-100 to a final concentration of 1% (v/v), mix well, and keep on ice.
  2. Incubate at 4 °C for 30-60 min.
  3. Centrifuge at 12,000 *× g* for 15 min at 4 °C.
  4. Remove upper layer of lipid and transfer the supernatant to a new 1.5-ml tube.
  5. Optional but recommended: Repeat Steps C3 and C4 for twice to get rid of maximum amount of lipid.
  6. Store the extracted samples at -80 °C until further BCA concentration measurement (summarized in Table 1).
    **Table 1.** Protein concentration from different fat pads

**sWAT**	Sample 1	Sample 2	Sample 3		Average
Concentration (μg/μl)	3.81	3.63	4.96		4.13
					
**eWAT**	Sample 1	Sample 2	Sample 3	Sample 4	Average
Concentration (μg/μl)	3.58	3.97	3.59	4.16	3.83
					
**BAT**	Sample 1	Sample 2	Sample 3	Sample 4	Average
Concentration (μg/μl)	8.23	8.62	7.48	8.00	8.08
  7. Perform Western Blotting (see Figure 3) or other applications.
    **Figure 3.**
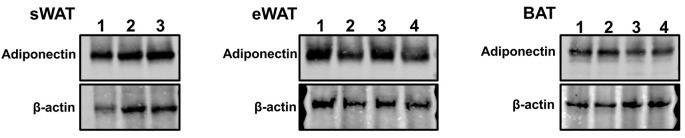
Representative Western blot images of protein samples extracted from AT. A total of 15 μg protein in each sample was loaded on an SDS-PAGE gel. Adiponectin and an internal control β-actin in sWAT (left), eWAT (middle) and BAT (right) were probed.

## Notes

1. An initial volume of RIPA buffer can be added to 0.6 ml/100 mg tissue. With larger volume of lysis buffer, it is easier to remove the lipid layer and thus achieve higher efficiency of depleting lipid.
2. For more representative Western blotting images, please refer to our cited publications (An *et al.*, 2017 and 2019).
3. This protocol is not applicable to specifically enrich adipose tissue membrane proteins.

## Recipes

1. RIPA buffer
  50 mmol/l Tris-HCl (pH 8.0)
  0.25 mol/l NaCl
  5 mmol/l EDTA
  1% Triton X-100 (v/v)
